# Death Following the Excision of a Nævus

**Published:** 1864-05

**Authors:** 


### DEATH FOLLOWING THE EXCISION OF A NÆVUS.

Dr. S. Cabot related to the Boston Society for Medical Improvement, the following case:

March 8th—A child a seven months and a few days old was brought to my office for advice, in reference to a vascular tumor, (raspberry,) situated on the right side of the os frontis, at some distance in front of the coronal suture. As it was increasing rapidly in size, I advised that it should be removed at once, and as it was situated over a smooth bony surface, favorable for the easy arrest of bleeding, I decided to excise, rather then to apply ligature. Assisted by my neighbor, Dr. J. C. White, who kindly attended to the etherization, I cut out the tumor by two semi-lunar incisions, extending through the scalp. There was but little bleeding, about an ounce—certainly not two ounces of blood were lost; two small vessels were tied, and the sides of the wound were brought together by uninterrupted silk sutures. A compress and bandage were applied, and the operation terminated. It took but a short time, and Dr. White says that not more than an ounce of ether was used.

I was obliged to leave my house as soon as the operation was finished, and did not return till after the patient had gone. I told the father of the child to call in a physician and have the stitches removed on the second day.

I should have thought no more about the case, as nothing about it gave any ground for anxiety, if I had not received, on the 14th, a letter from Dr. A. LeB. Monroe, dated 11th, saying, "The child from whose head you removed an erectile tumor last Tuesday, died the next day. I was called the next morning. Pulse very weak and frequent, skin cool, pupils dilated, conjunctivæ injected. At 3 P. M., pulse imperceptible, pupils more dilated, and unaffected by a strong light, respiration slow and difficult; died an hour or so after.

This morning made a *post-mortem* examination. Upon reflecting the scalp, the intervening cellular tissue was found infiltrated with blood, from an inch or more forward of the wound, back over the occiput to the nape of the neck. The track of blood varied from one to two inches in width. Upon removing the calvarium, the vessels of the brain were seen unusually distended, and the brain itself rather softer than we usually find it without disease. The mother says the child never recovered perfect consciousness; it nursed once on the way home, and once afterwards. About 12 P. M. it vomited, and after until 2 A. M." Dr. M. in a subsequent letter says: "The ligature you applied remained firm, and I found no artery unligated." Dr. M. told me afterward that there had been no escape of blood externally, the dressings were unstained.

On reviewing this case, we find a healthy child, after an operation involving a small surface of the head, (the wound when closed not being more than an inch in length,) accompanied by very moderate loss of blood and small degree of etherization, remains in a state of partial unconsciousness for twelve hours, then it is seized with vomiting, which lasts for two hours, followed by collapse and death in twelve hours more—the whole time from the operation to the death being about twenty-six hours.—*Boston Medical and Surgical Journal*, April 14, 1864.

